# Effects of Xylanase in Corn- or Wheat-Based Diets on Cecal Microbiota of Broilers

**DOI:** 10.3389/fmicb.2021.757066

**Published:** 2021-10-13

**Authors:** Jian Wang, Heng Cao, Chengling Bao, Yajing Liu, Bing Dong, Chunlin Wang, Zhenda Shang, Yunhe Cao, Suozhu Liu

**Affiliations:** ^1^State Key Laboratory of Animal Nutrition, College of Animal Science and Technology, China Agricultural University, Beijing, China; ^2^College of Animal Science, Tibet Agricultural and Animal Husbandry University, Linzhi, China

**Keywords:** xylanase, wheat, corn, broiler, cecal microbiota

## Abstract

Xylanase has been demonstrated to improve growth performance of broilers fed wheat- or corn-based diets due to its ability to degrade arabinoxylans (AX). However, content and structure of AX in corn and wheat are different, comparing effects of xylanase on cecal microbiota of broilers fed corn- or wheat-based diets could further elaborate the mechanism of the specificity of xylanase for different cereal grains. Thus, a total of 192 one-day-old broilers were randomly allotted into four dietary treatments, including wheat-soybean basal diet, wheat-soybean basal diet with 4,000U/kg xylanase, corn-soybean basal diet, and corn-soybean basal diet with 4,000U/kg xylanase to evaluate interactive effects of xylanase in corn- or wheat-based diets on broilers cecal microbiota during a 6-week production period. The results indicated that bacterial community clustering was mainly due to cereal grains rather than xylanase supplementation. Compared with broilers fed wheat-based diets, corn-based diets increased alpha-diversity and separated from wheat-based diets (*p*<0.05). Xylanase modulated the abundance of specific bacteria without changing overall microbial structure. In broilers fed wheat-based diets, xylanase increased the abundance of *Lactobacillus*, *Bifidobacterium*, and some butyrate-producing bacteria, and decreased the abundance of non-starch polysaccharides-degrading (NSP) bacteria, such as *Ruminococcaceae* and Bacteroidetes (*p*<0.05). In broilers fed corn-based diets, xylanase decreased the abundance of harmful bacteria (such as genus *Faecalitalea* and *Escherichia-Shigella*) and promoted the abundance of beneficial bacteria (such as *Anaerofustis* and *Lachnospiraceae_UCG_010*) in the cecum (*p*<0.05). Overall, xylanase supplementation to wheat- or corn-based diets improved broilers performance and cecal microbiota composition. Xylanase supplementation to wheat-based diets increased the abundance of butyrate-producing bacteria and decreased the abundance of NSP-degrading bacteria. Moreover, positive effects of xylanase on cecal microbiota of broilers fed corn-based diets were mostly related to the inhibition of potentially pathogenic bacteria, and xylanase supplementation to corn-based diets slightly affected the abundance of butyrate-producing bacteria and NSP-degrading bacterium, the difference might be related to lower content of AX in corn compared to wheat.

## Introduction

Xylanase has been widely applied in the broiler industry due to its ability to degrade arabinoxylans (AX) in poultry diets (Bautil et al., 2019). Corn and wheat are dominant feed grains for broilers diets; however, concentration and structure of AX in corn and wheat are different (Cowieson, 2010). At the present, most reports have focused on positive effects of xylanase on performance in broilers fed viscous wheat-based diets (Gonzalez-Ortiz et al., 2017; Liu and Kim, 2017; Wang et al., 2021). However, benefits of xylanase on growth performance of broilers supplemented non-viscous corn-based diets also were recorded (Khadem et al., 2016). Xylanase can reduce chyme viscosity, release nutrients from inside cell wall, and xylo-oligosaccharides (XOS) as a result of AX degradation in the gastrointestinal tract (GIT), which may then act like a prebiotic (Gonzalez-Ortiz et al., 2017; Petry et al., 2021). Previous studies have demonstrated that the dominant mode of action responsible for benefits of xylanase in corn- or wheat-based diets may be distinct and impacts of xylanase on growth performance, nutrient utilization, digesta characteristics, and cecal volatile fatty acid (VFA) concentrations of broilers fed corn- or wheat-based diets were compared (Kiarie et al., 2014; Munyaka et al., 2016; McCafferty et al., 2019a,b).

Cecal microbiota has the most complex microbiota in GIT which was associated with the health of broilers (Yadav and Jha, 2019). The GIT microbiota is shaped by both the environment and the broiler itself (Liu et al., 2021). Environmental changes, especially dietary factors, such as feed components and additives, have been widely demonstrated to regulate GIT microbiota (Shang et al., 2018). Cecal VFA concentrations are considered to be associated with bacterial fermentation in the large intestine (Ríos-Covián et al., 2016). Xylanase changed VFA concentrations in broilers cecum when the diets were corn- or wheat-based (McCafferty et al., 2019a,b). We hypothesized that supplemental xylanase might exert positive effects on cecal microbiota of broilers fed corn- or wheat-based diets by different gut bacterial communities.

16S rRNA gene sequencing analysis is a tool that has been widely applied to evaluate the diversity of GIT microbiota (Shang et al., 2018). To further elaborate the specificity of xylanase for feed ingredients, and the impacts of xylanase on cecal microbiota, we conducted the following experiment by feeding broilers corn- or wheat-based diets for a 6-week period and assessed the bacterial composition by 16S rRNA gene sequencing.

## Materials and Methods

The present study was approved by the Laboratory Animal Welfare and Animal Experimental Ethical Inspection Committee of China Agricultural University (Beijing, China).

### Preparation of Xylanase

The xylanase (2,000,000U/kg) used in this experiment was preserved by our laboratory. An acidic xylanase gene (*xynA*) cloned from *Aspergillus sulphureus* was coden-optimized (GenBank accession number: EF114744) and expressed in *Pichia pastoris* (Cao et al., 2007). A disulfide bridge and proline residue substitutions were introduced to create xylanase mutants (AT-xynA) aiming to improve the thermostability of AT-xynA. Moreover, construction of a double-copy expression strain increased AT-xynA produced in *Pichia pastoris* (Wang et al., 2020). AT-xynA exhibited a resistance to an acidic environment (over 70% residual activity remained after 120min incubation at pH 2.2) and was stable at high temperature (about 50% residual activity remained after 9.5min incubation at 70°C; Yang et al., 2017a). High-density fermentation of the xylanase was processed in a 15-L fermenter as previously described (Yang et al., 2017a). The actual xylanase product was obtained by mixing 66% of the liquid fermentation broth produced above with 34% soybean meal and then air dried for 24h. This resulted in a xylanase preparation containing approximately 2,000,000units (U) of xylanase per kg. One unit of xylanase activity is defined as the amount of enzyme releasing 1μmol of xylose per min under pH 3.0 and 50°C (Yang et al., 2017a).

### Experiment Animals, Management, Design, and Diets

Individually numbered metal tags were attached to the wings of 192 1-day-old Arbor Acres broilers (46.14±0.42g) for individual identification. Broilers were vaccinated with Newcastle disease vaccine on d 7 and 28, and vaccinated with an inactivated infectious bursal disease vaccine on d 14 and 21. The broilers were raised in an environmentally controlled room, and they were exposed to constant light. The initial room temperature for 1-day-old broilers was controlled at about 33°C, and the temperature reduced 3°C/week until 24°C as the age of broilers increased. Broilers were randomly assigned to one of four diet treatments, and each treatment had six replicates with eight broilers. Broilers were fed an early phase diet (d 1 to 21) and a late phase diet (d 21 to 42). Dietary treatments consisted of 2×2 factorial design including two cereals (corn and wheat) and two enzyme treatments (control and xylanase): (1) wheat-soybean-based diet (WCON), (2) wheat-soybean-based diet with 4,000U/kg xylanase (WXY), (3) corn-soybean-based diet (CCON), and (4) corn-soybean-based diet with 4,000U/kg xylanase (CXY). The composition of experimental diets was shown (Table 1), and nutrient levels of diets satisfied National Research Council (National Research Council, 1994).

**Table 1:** Composition and nutrient levels of basal diets (%, as fed basis).

Items	1 to 21 d of age		22 to 42 d of age		Corn	Wheat	Corn	Wheat
**Ingredients**
Corn	61.15	–	67.99	–
Wheat	–	62.16	–	67.28
Soybean meal, 43% crude protein	28.00	25.80	22.00	19.47
Fish meal, 64.6% crude protein	3.71	3.46	2.60	4.62
Soybean oil	3.00	4.96	3.60	5.56
Dicalcium phosphate	1.28	1.02	1.38	0.83
Limestone	1.26	1.40	1.10	1.16
Salt	0.30	0.30	0.30	0.30
L-lysine HCl, 78%	–	–	0.12	–
DL-Methionine, 98%	0.15	0.14	0.07	0.02
L-Threonine, 98%	0.04	0.01	0.09	0.01
Chromic oxide	0.25	0.25	0.25	0.25
Vitamin-mineral premix^1^	0.50	0.50	0.50	0.50
Total	100.00	100.00	100.00	100.00
**Calculated nutrient content^2^**				
Metabolizable energy, kcal/kg	3,052	3,050	3,157	3,150
Crude protein	20.08	20.95	19.37	20.36
Calcium	1.00	1.00	0.90	0.90
Available phosphorous	0.45	0.45	0.35	0.35
Digestible lysine	0.86	0.86	0.73	0.73
Digestible methionine	0.30	0.30	0.28	0.28
Digestible threonine	0.63	0.63	0.56	0.56

1 *Vitamin A, 11,000IU; vitamin D, 3,025IU; vitamin E, 22mg; vitamin K_3_, 2.2mg; thiamine, 1.65mg; riboflavin, 6.6mg; pyridoxine, 3.3mg; cobalamin, 17.6μg; nicotinic acid, 22mg; pantothenic acid, 13.2mg; folic acid, 0.33mg; biotin, 88μg; choline chloride, 500mg; iron, 48mg; zinc, 96.6mg; manganese, 101.76mg; copper, 10mg; selenium, 0.05mg; iodine, 0.96mg; and cobalt, 0.3mg*.

2 *Values were calculated according to the National Research Council (1994)*.

### Sample Collection and Processing

Average daily gain (ADG), average daily feed intake (ADFI), and feed conversion ratio (FCR) of broilers from d 1 to 42 were calculated based on body weight and feed intake. One broiler from each replicate was slaughtered on d 14, 28, and 42 for collection of cecal digesta. The cecal digesta was snap-frozen in liquid nitrogen and then stored at −80°C.

### Cecal Microbiota Analysis

For microbial diversity analysis, total genomic DNA from cecal digesta samples was extracted by using the EZNA stool DNA kit (Omega Biotek, Norcross, GA). The concentration of DNA was quantified by a NanoDrop 2000 spectrophotometer (Thermo Scientific, MA, United States), and the quality of DNA was analyzed by electrophoresis with 2% agarose gels. Then, the V3-V4 region of the 16S rRNA gene was amplified using genomic DNA of cecal digest samples as the template, and 338F (5'barcodeACTCCTACGGGAGGCAGCAG3') and 806R (5'GGACTACHVGGGTW TCTAAT3') as the primers. The PCR reaction conditions were as: initial denaturation at 95°C for 3min, then 27cycles of 95°C for 30s, 55°C for 30s and 72°C for 45s, and finally 72°C for 5min. The PCR products were purified by the AxyPrep DNA Gel Extraction Kit (Axygen Biosciences, Union City, CA). The purified amplicons were pooled in equimolar concentrations and then sequenced on Illumina MiSeq platform. Trimmomatic (version 3.29) was applied to quality-filter raw Illumina fastq files. Operational taxonomic units (OTUs) were clustered with a similarity of 97% using UPARSE (version 7.0).^1^ The raw data have been submitted to the NCBI Sequence Read Archive (SRA) database (PRJNA753623).

### Statistical Measurements

To determine interaction effects of cereal type and xylanase supplementation on growth performance and microbiota alpha-diversity of broilers, a two-way ANOVA was performed using SAS (version 9.2, 2008). Each treatment contained six pens, and the pen was considered as an experiment unit. *p*<0.05 was considered significant, and 0.05≤*p*≤0.10 was regarded a trend. When a significant effect was observed, the data were further analyzed by one-way ANOVA (version 9.2, 2008). The rarefaction curves of OTUs were calculated by QIIME to determine the sequencing coverage across samples. Alpha-diversity was calculated by Sobs (the observed OTUs), Chao (the Chao 1 estimator), Ace (the ACE estimator), and PD (Phylogenetic diversity). Principal coordinate analysis (PCoA) on Weighted UniFrac distance was conducted. Analysis of similarity (ANOSIM) with 999 permutations was used to assess the similarity between bacterial community structure by measuring *R* value (between 0 and 1). *R*=0 indicates that two groups are similar, and *R*=1 suggests two groups are different. The linear discriminant analysis effect size (LEfSe) method was applied for the analysis of significant differences in abundance of microbiota community.

## Results

### Growth Performance

**Figure 1:**
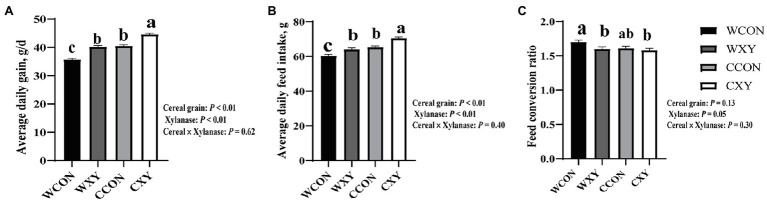
Growth performance of broilers fed corn- or wheat-based diets with or without xylanase during 6-week period. Dietary treatments consisted of wheat-soybean basal diet (WCON), wheat-soybean basal diet with 4,000U/kg xylanase (WXY), corn-soybean basal diet (CCON), and corn-soybean basal diet with 4,000U/kg xylanase (CXY). **(A)** Average daily gain. **(B)** Average daily feed intake. **(C)** Feed conversion ratio. Significant differences (*p*<0.05) among different groups are indicated by different letters. Values are expressed as means ± SEM.

The ADG, ADFI, and FCR of broilers from different dietary treatments were shown in Figure 1. From 1 to 42 d of age, ADG and ADFI of broilers from WXY and CCON groups were higher than broilers from WCON group (*p*<0.05); however, ADG and ADFI of broilers from CXY group were higher compared with broilers from WXY to CCON groups (*p*<0.05). Broilers from WCON group exhibited higher FCR compared to broilers from WXY and CXY groups (*p*<0.05), and FCR of broilers from CCON group was similar to those from WCON, WXY, and CXY groups (*p*>0.05). Additionally, cereal main effect influenced ADG and ADFI (*p*<0.05), the broilers receiving corn-based diets showed higher ADG and ADFI than those fed wheat-based diets (*p*<0.05). Xylanase main effect also influenced ADG and ADFI (*p*<0.05), and tended to influence FCR (*p*=0.05), xylanase supplementation to broilers diets could improve ADG, ADFI, and FCR of broilers (*p*<0.05). However, no interactive effects of cereal type and xylanase were observed on broilers growth performance.

### Cecal Microbiota Analysis

**Figure 2:**
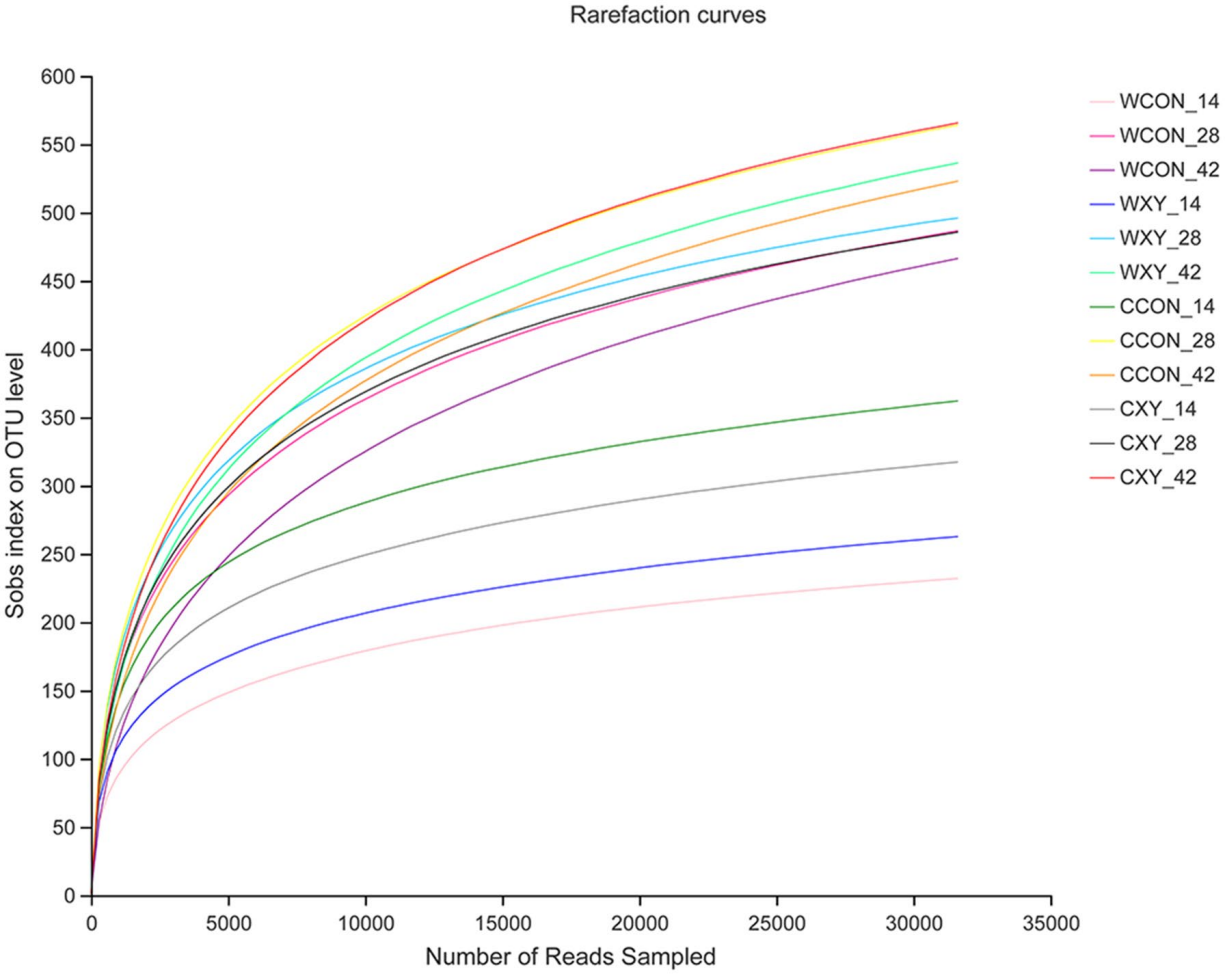
Rarefaction curves showing sequence coverage across samples on the observed number of OTUs. For rarefaction plots, calculated based on the observed OTUs to determine the sequencing coverage across samples. OTUs, operational taxonomic units. Wheat-soybean basal diet (WCON); wheat-soybean basal diet with 4,000U/kg xylanase (WXY); corn-soybean basal diet (CCON); and corn-soybean basal diet with 4,000U/kg xylanase (CXY).

In this experiment, a total of 3,211,886 high-quality sequence reads of samples, with the average read length of 412 base (bp), were obtained for subsequent analysis. In total, 1,146 distinct OTUs with a 97% identity were generated that fell into 13 phyla, 24 classes, 44 orders, 70 families, and 175 genera. Rarefaction curves based on the observed OTUs approached asymptotic for each dietary treatment on d 14, 28, and 42, indicating the availability of sufficient numbers of OTUs to represent each microbiota community, and age-linked increasing trend in the number of observed OTUs was observed in broilers (Figure 2).

**Figure 3:**
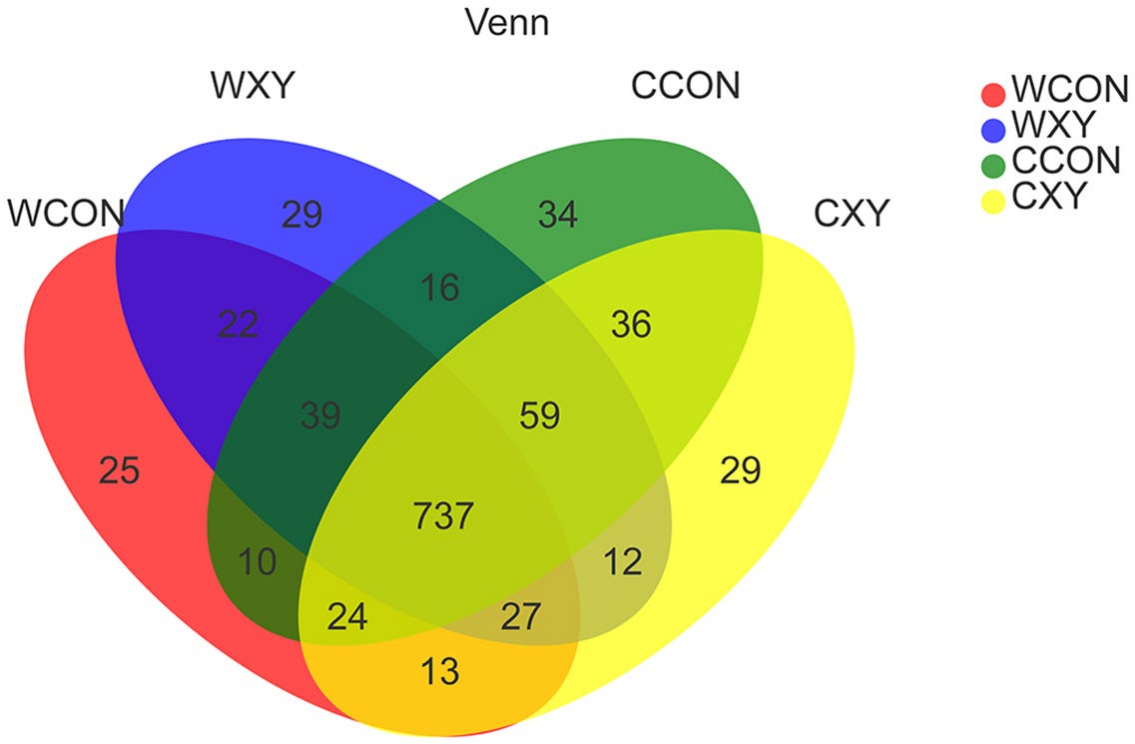
Bacterial OTU community composition of the broilers fed corn- or wheat-based diets. Venn diagrams were generated to make qualitative comparisons of the core and unique OTUs among WCON, WXY, CCON, and CXY groups. OTUs, operational taxonomic units. Wheat-soybean basal diet (WCON); wheat-soybean basal diet with 4,000U/kg xylanase (WXY); corn-soybean basal diet (CCON); and corn-soybean basal diet with 4,000U/kg xylanase (CXY).

**Figure 4:**
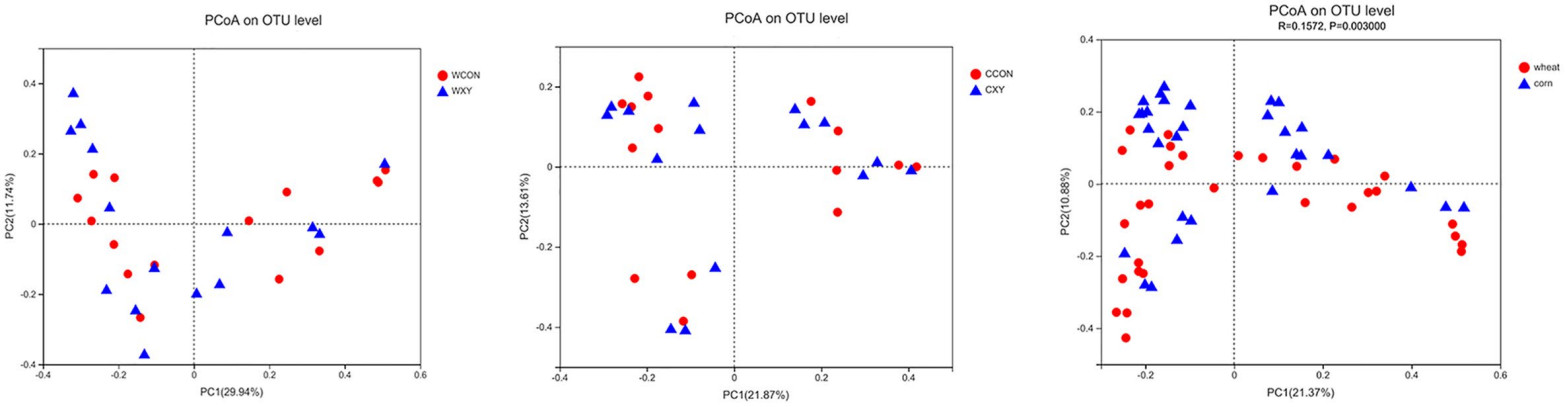
Principal coordinate analysis (PCoA) representing similarity of cecal microbiota in broilers fed corn- or wheat-based diets. The PCoA was performed by using Weighted UniFrac distance to determine β-diversity of bacterial communities between different groups. No distinct clustering due to xylanase supplementation was denoted (WCON vs. WXY; CCON vs. CXY). However, a less pronounced but distinct variation was observed in broilers fed wheat- or corn-based diets (ANOSIM, *R*=0.1572, *P*=0.003). Wheat-soybean basal diet (WCON); wheat-soybean basal diet with 4,000U/kg xylanase (WXY); corn-soybean basal diet (CCON); and corn-soybean basal diet with 4,000U/kg xylanase (CXY).

Venn diagrams were generated to make qualitative comparisons of the core and unique genera between WCON, WXY, CCON, and CXY groups (Figure 3). The core OTUs of the four treatments were 737, and the number of unique genera for WCON, WXY, CCON, and CXY groups was 25, 29, 34, and 29, respectively. As shown in Table 2, there was not a significant interaction between cereal type and xylanase supplementation in cecal microbiota alpha-diversity of broilers (*p*>0.05). On d 14, cereal main effect influenced indices of Sobs, Ace, Chao (*p*<0.05), and PD (*p*=0.08), and the broilers fed corn-based diets exhibited higher Sobs, Ace, Chao, and PD values compared to those receiving wheat-based diets (*p*<0.05). On d 42, PD values were also significantly affected by the cereal type (*p*<0.05), and broilers from CXY exhibited significantly higher PD values compared with broilers from WCON to WXY groups (*p*<0.05). The PCoA was performed by using Weighted UniFrac distance to determine β-diversity of microbial communities between different groups (Figure 4). Although the addition of xylanase in wheat- or corn-based diets improved growth performance of broilers, no distinct clustering due to xylanase supplementation was denoted. However, PCoA of the OTU data for chickens fed wheat- or corn-based diets revealed a less pronounced but distinct variation between cereal grains (ANOSIM, *R*=0.1572, *p*=0.003).

**Table 2:** Effects of xylanase on alpha-diversity of cecal bacterial community in broilers fed wheat- or corn-based diets^1^.

	WCON	WXY	CCON	CXY	SEM	Analysis of Variance		
						Cereal grain × Xylanase	Cereal grain	Xylanase
**d 14**
The observed OTUs	232.00^c^	262.67^bc^	362.00^a^	317.33^ab^	24.46	0.16	< 0.01	0.78
The ACE estimator	271.49^b^	311.52^ab^	420.13^a^	368.80^ab^	32.44	0.20	0.01	0.87
The Chao^1^ estimator	276.91^b^	315.58^b^	422.40^a^	374.52^ab^	34.35	0.16	0.01	0.68
Phylogenetic diversity	17.63	22.61	28.00	24.90	3.15	0.24	0.08	0.77
**d 28**
The observed OTUs	486.67	496.00	564.00	485.67	35.22	0.25	0.37	0.36
The ACE estimator	579.62	567.51	667.31	569.31	29.94	0.19	0.17	0.10
The Chao^1^ estimator	581.69	564.43	686.76	570.28	34.81	0.19	0.15	0.10
Phylogenetic diversity	32.74	32.99	35.40	32.96	1.92	0.50	0.51	0.58
**d 42**
The observed OTUs	466.33	536.33	523.00	565.67	32.98	0.69	0.23	0.13
The ACE estimator	588.28	648.24	645.53	655.60	41.29	0.56	0.46	0.42
The Chao^1^ estimator	598.90	662.40	667.68	656.46	43.40	0.41	0.49	0.57
Phylogenetic diversity	32.33^b^	34.91^b^	35.74^ab^	42.11^a^	2.05	0.38	0.03	0.10

1 *Alpha diversity analysis for bacterial community determined by 16s RNA sequencing. OTUs, operational taxonomic units. Wheat-soybean basal diet (WCON); wheat-soybean basal diet with 4,000U/kg xylanase (WXY); corn-soybean basal diet (CCON); and corn-soybean basal diet with 4,000U/kg xylanase (CXY)*.

**Figure 5:**
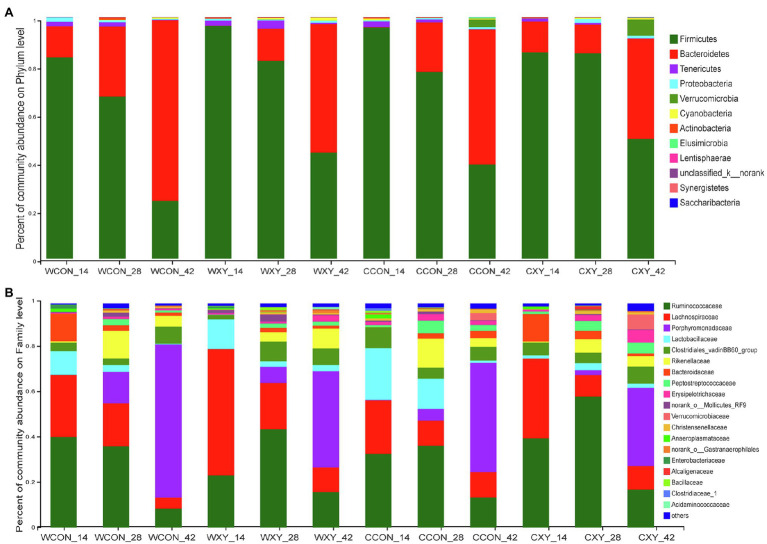
Effects of xylanase on cecal bacteria communities of broilers at phylum and family level. **(A)** Cecal bacterial community at the phylum level in broilers from four treatments (d 14, d 28, and d 42). **(B)** Cecal bacterial community at the family level with the relative abundance higher than 0.1% in broilers from four treatments (d 14, d 28, and d 42). Wheat-soybean basal diet (WCON); wheat-soybean basal diet with 4,000U/kg xylanase (WXY); corn-soybean basal diet (CCON); and corn-soybean basal diet with 4,000U/kg xylanase (CXY).

To further elaborate effects of xylanase supplementation to wheat- or corn-basal diets on broilers cecal microbiota, we then compared the bacterial community at phylum and family levels across four dietary treatments at each sampling time point. At the phylum level (Figure 5A), microbiota of cecal microbiota community was dominated by Firmicutes, Bacteroidetes, Proteobacteria, Tenericutes, and Actinobacteria independent of sampling time point and xylanase supplementation. Two major phyla (Firmicutes and Bacteroidetes) accounted for more than 90% of the cecal bacterial community at each time point in four treatments. The other phyla, such as Proteobacteria, Tenericutes, and Actinobacteria, were represented at relatively low abundance. Bacteroidetes increased in abundance with time in four dietary treatments; however, the relative abundance of Firmicutes decreased with age. At the family level (Figure 5B), *Ruminococcaceae*, *Lachnospiraceae*, *Clostridiales_vadinBB60_group*, *Lactobacillaceae*, *Peptococcaceae*, and *Erysipelotrichaceae* assigned to Firmicutes phyla, *Porphyromonadaceae*, *Rikenellaceae*, and *Bacteroidaceae* assigned to Bacteroidetes phyla, and *norank_o__Mollicutes_RF9* which belongs to Tenericutes phyla were dominant families in the cecum. At 14days of age, *Ruminococcaceae* was the most abundant family in WCON, CCON, and CXY groups, and *Lachnospiraceae* was the most abundant family in WXY group. At 28days of age, *Ruminococcaceae* was the most abundant family, and at 42days of age, *Porphyromonadaceae* was the dominant family in four dietary treatments.

**Figure 6:**
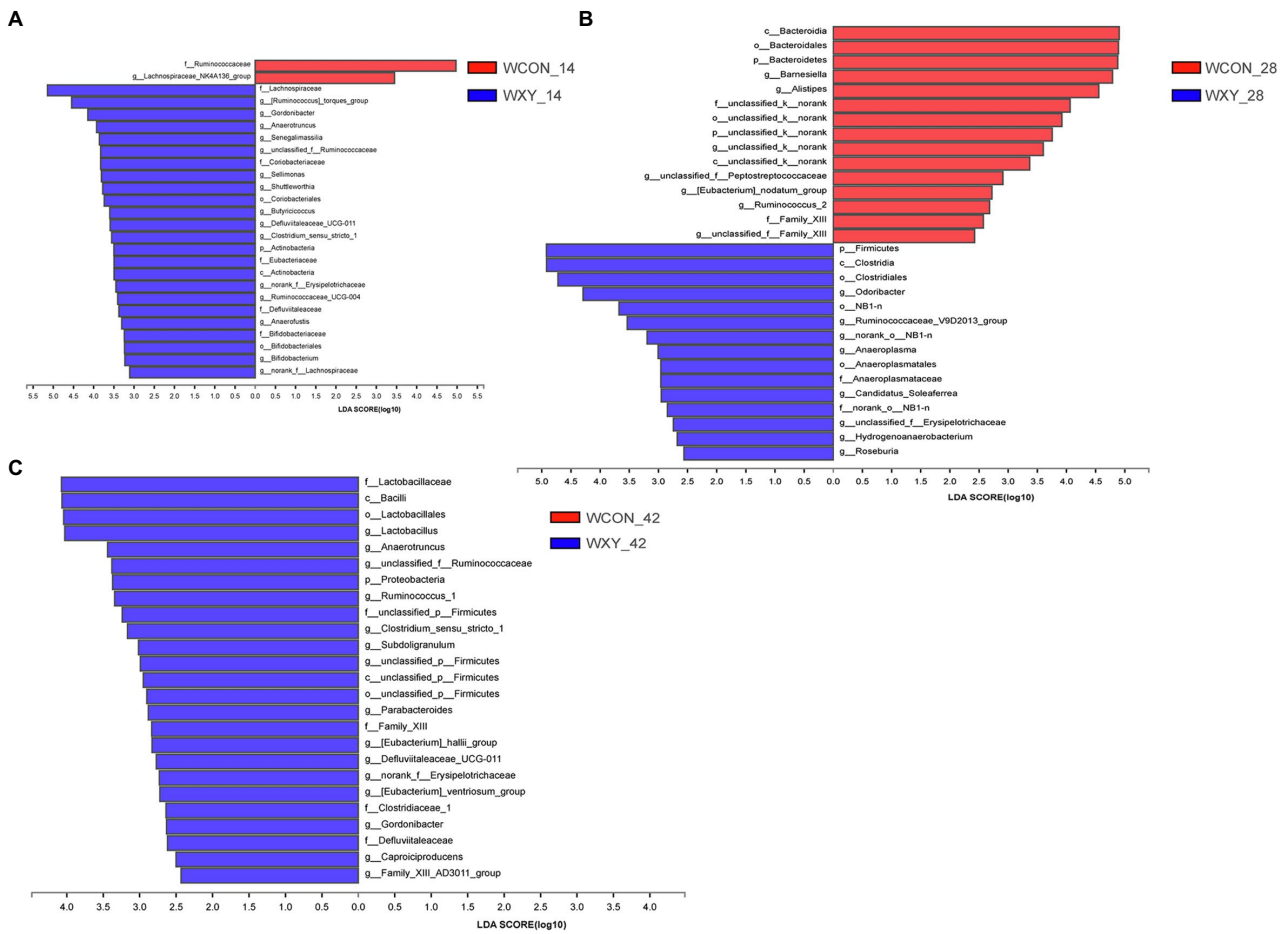
The LEfSe analysis for cecal bacteria communities from phylum to genus level in WCON and WXY groups. Different bacterial communities from phylum to genus level between WCON (denoted by red bars) and WXY (denoted by blue bars) groups on d 14 **(A)**, d 28 **(B)**, and d 42 **(C)**. Wheat-soybean basal diet (WCON); wheat-soybean basal diet with 4,000U/kg xylanase (WXY).

**Figure 7:**
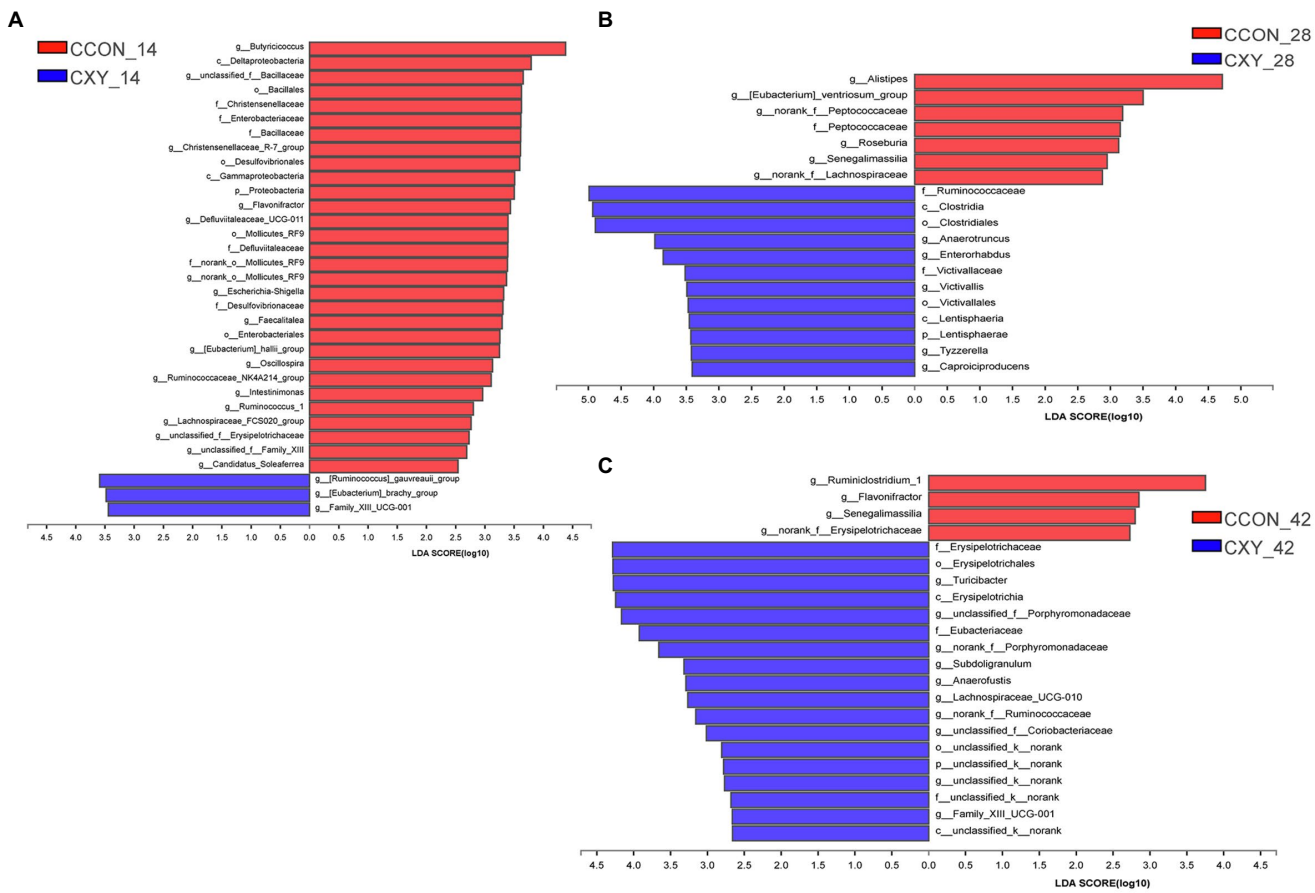
The LEfSe analysis for cecal bacteria communities from phylum to genus level in CCON and CXY groups. Different bacterial communities from phylum to genus level between CCON (denoted by red bars) and CXY (denoted by blue bars) groups on d 14 **(A)**, d 28 **(B)**, and d 42 **(C)**. Corn-soybean basal diet (CCON); corn-soybean basal diet with 4,000U/kg xylanase (CXY).

To further probe how AT-xynA modulated GIT microbial community, alterations in cecal microbiota were also analyzed by the LefSe method on d 14, 28, and 42. On d 14 (Figure 6A), in WCON and WXY groups, the abundance of genus *norank_f_Lachnospiraceae*, *Shuttleworthia*, *Ruminococcus_torques_group*, and *Sellimonas* (family *Lachnospiraceae*) was higher in WXY group (*p*<0.05). The abundance of genus *Ruminococcaceae_UCG_004*, *unclassified_f_Ruminococcaceae*, *Anaerotruncus*, and *Butyricicoccus* (family *Ruminococcaceae*) was also higher in WXY group, while the abundance of family *Ruminococcaceae* was higher in WCON group (*p*<0.05). The relative abundance of genus *Defluviitaleaceae_UCG_011* (family *Defluviitaleaceae*), *Clostridium_sensu_stricto_1*, *Anaerofustis* (family *Eubacteriaceae*), and *norank_f__Erysipelotrichaceae* was higher in WXY group (*p*<0.05). The phylum Actinobacteria, order *Coriobacteriales*, and genus *Bifidobacterium*, *Gordonibacter*, and *Senegalimassilia* were also higher in WXY group (*p*<0.05). On d 14 (Figure 7A), in CCON and CXY groups, higher relative abundance of *Ruminococcus__gauvreauii_group*, *Eubacterium__brachy_group*, and *Family_XIII_UCG_001* was observed in CXY group (*p*<0.05). The abundance of genus *Lachnospiraceae_FCS020_group*, *Faecalitalea*, *Eubacterium__hallii_group*, *Flavonifractor*, *Intestinimonas*, *Candidatus_Soleaferrea*, and *Ruminococcaceae_NK4A214_group* was higher in CCON group (*p*<0.05). The abundance of genus *Christensenellaceae_R_7_group* (family *Christensenellaceae*), *Defluviitaleaceae_UCG_011* (family *Defluviitaleaceae*), *unclassified_f__Bacillaceae* (order *Bacillales*), and *norank_o__Mollicutes_RF9* (order *Mollicutes_RF9*) was increased in CCON group (*p*<0.05). The abundance of phylum Proteobacteria including family *Enterobacteriaceae* and family *Desulfovibrionaceae* was also higher in CCON group (*p*<0.05).

On d 28 (Figure 6B), in WCON and WXY groups, the phylum Firmicutes were higher in WXY group, while the phylum Bacteroidetes were higher in WCON group (*p*<0.05). The genus *Anaeroplasma* belonged to order *Anaeroplasmatales* and the genus *Odoribacter*, *Hydrogenoanaerobacterium*, and *Ruminococcaceae_V9D2013_group* were higher in WXY group (*p*<0.05). On d 28 (Figure 7B), in CCON and CXY groups, the abundance of class *Clostridia* including genus *Anaerotruncus*, *Tyzzerella*, and *Caproiciproducens*, as well as the phylum Lentisphaerae, was higher in CXY group (*p*<0.05). The abundance of genus *Alistipes*, *Eubacterium__ventriosum_group*, *norank_f__Peptococcaceae*, *Senegalimassilia*, and *norank_f__Lachnospiraceae* was higher in CCON group (*p*<0.05).

On d 42 (Figure 6C), in WCON and WXY groups, the abundance of genus *Eubacterium_hallii_group*, *Eubacterium_ventriosum_group*, *Gordonibacter*, and *Parabacteroides* was higher in WXY group (*p*<0.05). The abundance of genus *Anaerotruncus*, *unclassified_f_Ruminococcaceae*, *Subdoligranulum*, and *Caproiciproducens* (family *Ruminococcaceae*) was also higher in WXY group (*p*<0.05). Higher abundance of the genus *Clostridium_sensu_stricto_1* belonging to family *Clostridiaceae_1*, genus *Defluviitaleaceae_UCG_011* belonging to family *Defluviitaleaceae*, and genus *Lactobacillus* from class *Bacilli* was observed in WXY group (*p*<0.05). On d 42 (Figure 7C), in CCON and CXY groups, the abundance of class *Erysipelotrichia*, genus *Lachnospiraceae_UCG_010*, *Subdoligranulum*, *Anaerofustis*, and *norank_f_Runmiococcaceae* was higher in CXY group, while the abundance of genus *Ruminiclostridium_1*, *Senegalimassilia*, *Flavonifractor*, and *norank_f__Erysipelotrichaceae* was higher in CCON group (*p*<0.05).

**Figure 8:**
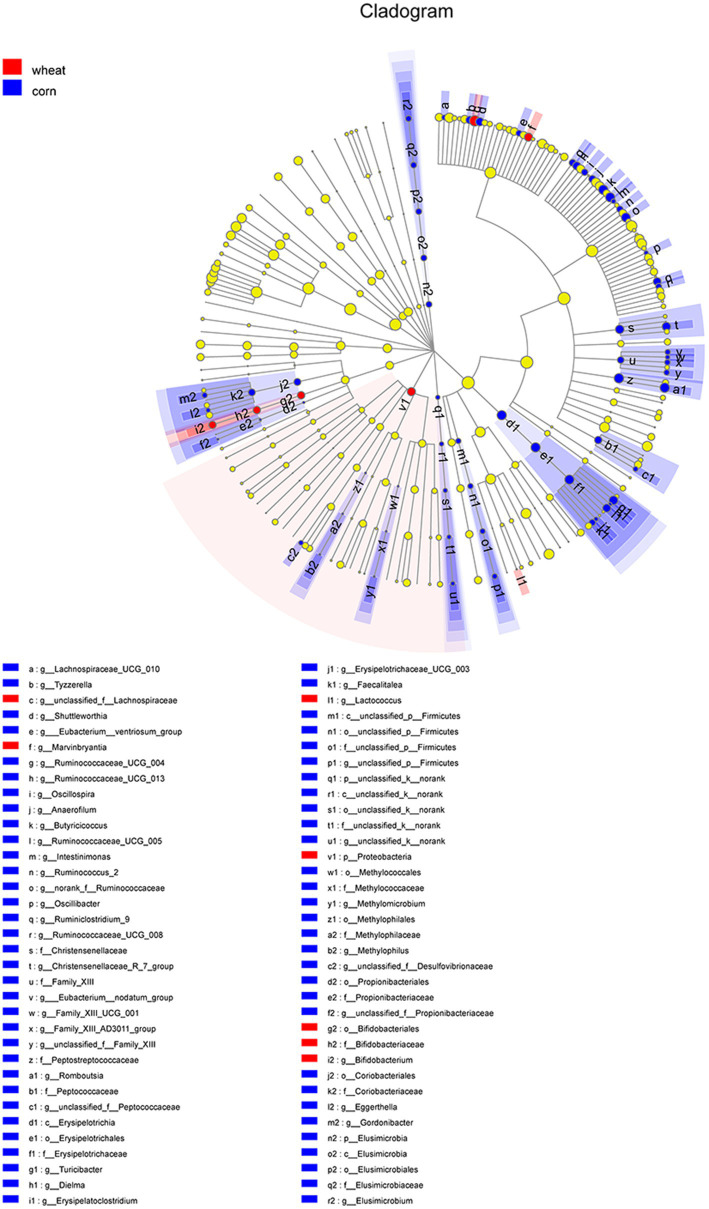
The LEfSe analysis for cecal bacteria communities from phylum to genus level in broilers fed wheat- or corn-based diets. The circles from inner to outer represent distinct bacteria from phylum to genus level, respectively. The yellow dots inserted in the circle demonstrated no significant differences in bacteria communities between two dietary treatments.

The cecal microbiota of broilers fed corn- or wheat-based diets was also compared by the LefSe method (Figure 8). The abundance of genus *Lachnospiraceae_UCG_010*, *Tyzzerella*, *Shuttleworthia*, *Eubacterium__ventriosum_group*, *Ruminococcaceae_UCG_013*, *Ruminococcaceae_UCG_004*, *Oscillospira*, *Anaerofilum*, *Butyricicoccus*, *Ruminococcaceae_UCG_008*, *Ruminococcaceae_UCG_005*, *Intestinimonas*, *Ruminococcus_2*, *norank_f__Ruminococcaceae*, *Oscillibacter*, and *Ruminiclostridium_9* was higher in broilers fed corn-based diets (*p*<0.05). The abundance of genus *Christensenellaceae_R_7_group* (*Christensenellaceae* family), *Romboutsia* (*Peptostreptococcaceae* family), genus *unclassified_f__Propionibacteriaceae* (*Peptococcaceae* family), *Erysipelatoclostridium*, *Turicibacter*, *Dielma*, *Erysipelotrichaceae_UCG_003*, *Faecalitalea* (family *Erysipelotrichaceae*), *Elusimicrobium* (phylum Elusimicrobia), *unclassified_f__Peptococcaceae* (family *Propionibacteriaceae)*, *Eggerthella*, *Gordonibacter* (family *Coriobacteriaceae*), *Methylomicrobium* (family *Methylococcaceae*), and *Methylophilus* (family *Methylophilaceae*) was higher in broilers receiving corn-based diets (*p*<0.05). Moreover, higher abundance of the phylum Proteobacteria, order *Bifidobacteriales*, family *Bifidobacteriaceae*, genus *unclassified_f__Lachnospiraceae*, *Marvinbryantia*, *Bifidobacterium*, and *Lactococcus* was observed in broilers receiving wheat-based diets (*p*<0.05).

## Discussion

In the current study, broilers receiving corn-based diets showed higher ADG and ADFI than broilers supplemented wheat-based diets. Masey-O'Neill et al. (2014) also reported that feeding broilers with corn-based diets increased body weight gain and feed intake compared to broilers fed wheat-based diets from d 1 to 28. This observation might be related to the higher concentration of soluble NSP in viscous wheat. The soluble NSP is known to exhibit antinutritive effects by increasing intestinal digesta viscosity, affecting gut transit time, thus impair nutrient absorption and utilization of broilers (Kiarie et al., 2014). However, similar to our results, it has been reported that feeding broilers with wheat-based diets supplemented with xylanase could improve growth performance, the improvement might be associated with the alleviation of detrimental effects of soluble NSP on digesta viscosity (Gonzalez-Ortiz et al., 2017; Liu and Kim, 2017). Moreover, in this study, xylanase improved growth performance of broilers receiving corn-based diets. Benefits of xylanase on performance of broilers fed non-viscous corn-based diets also have been reported, indicating that there are other mechanisms besides this mode of viscosity reduction (Khadem et al., 2016). The primary mode of action may depend on the type of cereal grain, thus 16S rRNA gene high-throughput sequencing in this study was used to elaborate effects of xylanase on cecal microbiota of broilers supplemented corn- or wheat-based diets on d 14, 28, and 42.

The generated rarefaction curves have reached plateau with the Sobs index, suggesting that 16S rRNA sequencing libraries could represent most of OTUs present in all the samples sequenced in this study. As shown in Venn diagram, the number of OTUs in cecal microbiota among four dietary treatments was similar, and most of them shared. When comparing effects of xylanase on cecal microbiota alpha-diversity of broilers fed wheat- or corn-based diets, there was not a significant interaction between cereal type and xylanase supplementation in alpha-diversity, and xylanase main effect also did not affect alpha-diversity of broilers. However, we found that broilers receiving corn-based diets had richer and more diverse microbiota than broilers fed wheat-based diets on d 14 and 42, indicating that microbiota of broilers receiving corn-based diets exhibited higher richness. The increase in alpha-diversity has been reported to be beneficial to the health of broilers, the increased diversity might be able to promote the stability of microbiota (Díaz Carrasco et al., 2018; Dhillon et al., 2019). The analysis of PCoA based on dietary treatments did not exhibit distinct clustering patterns, the composition of GIT microbiota between WCON and WXY groups was similar, and beta diversity was also not different between CCON and CXY groups. However, the phylogenetic distances clearly revealed that type of cereal grain affected beta diversity, suggesting a distinction in microbial community structure of broilers receiving corn-based diets relative to broilers receiving wheat-based diets.

To further probe how xylanase modulates GIT microbial community of broilers receiving wheat- or corn-based diets, the variations in relative abundance of microbiota were analyzed. The development of GIT microbiota in broilers might be a continuous process of microbial community succession. In the current study, the development of microbiota community from an immature phase to a mature phase in four dietary treatments was clearly shown. As previously reported, cecal communities for all the broilers were dominated by members of Firmicutes and Bacteroidetes phylum (Ilaria et al., 2020). Firmicutes accounted for most of the cecal microbiota in young broilers, while Bacteroidetes were only enriched at the end of productive life. Butyrate-producing bacteria mostly belong to Firmicutes phylum (Chakraborti, 2015; Dai et al., 2021). Representatives of Bacteroidetes phylum mainly degrade polysaccharides producing propionate (Yang et al., 2013; Polansky et al., 2016). In quickly growing, young birds, butyrate is highly required to provide energy for the growth of intestinal cells, while adult birds have fully developed intestines and need less amount of butyrate (Petra et al., 2014; Onrust et al., 2015). Higher abundance of Bacteroidetes in adult broilers represented an effective balance between energy obtained from available nutrients and sustained growth (Ocejo et al., 2019). The alteration of the proportion of Firmicutes and Bacteroidetes indicates the change of produced butyrate in broilers cecum, thus can explain the results in this study that Bacteroidetes gradually increased throughout the production period.

When comparing cecal microbiota of broilers fed wheat-based diets, compared with WXY, higher abundance of family *Ruminococcaceae* on d 14 and phylum Bacteroidetes on d 28 was observed in WCON group. *Ruminococcaceae* has been reported to be related to cellulose-degrading capacity, and *Ruminococcaceae* is enriched in genes encoding for endo-1,4-beta-xylanase and cellulase (Biddle et al., 2013; Dai et al., 2020). Phylum Bacteroidetes also contain a high number of genes encoding for glycose hydrolases, such as β-xylosidase, endo-1,4-β-xylanase, and α-N-arabinofuranosidase, which makes Bacteroidetes become the primary phylum for the degradation of complex polysaccharides in GIT (Zhang et al., 2018a). Furthermore, it has been demonstrated that *Bacteroides* of the phylum Bacteroidetes was related to AX degradation (Zhang et al., 2018a,b). Dietary supplementation of xylanase to broilers could effectively hydrolyze AX in small intestines, thereby reducing the availability of polysaccharides to *Ruminococcaceae* and Bacteroidetes in the cecum. However, the abundance of microbiota associated with arabinoxylan-degrading capacity was not significantly different between broilers from CCON to CXY groups, we speculated that the difference might be related to the lower contents of arabinoxylans in corn.

Additionally, compared with WXY, the increased abundance of *Ruminococcaceae* in broilers from WCON group on d 14, and compared with CXY, higher abundance of order *Mollicutes_RF9* in CCON group on d 14 was observed. *Ruminococcaceae* has been demonstrated to be sensitive to starch, increased abundance of *Ruminococcaceae* in broilers from WCON group might also be related to lower ileal starch digestibility, because the addition of xylanase has been reported to increase starch digestibility (Choct et al., 1999; McCafferty et al., 2019a). The metagenome-assembled genomes of *Mollicutes_RF9* group also have the CAZyme related with starch degradation, thus lower concentration of undigested starch flowing into the cecum might limit the growth of *Mollicutes_RF9* (Ma et al., 2019).

Compared to WCON, higher abundance of family *Lachnospiraceae* (containing *norank_f_Lachnospiraceae*, *Shuttleworthia*, *Ruminococcus_torques_group*, and *Sellimonas*) on d 14, phylum Firmicutes, genus *Hydrogenoanaerobacterium*, and *Ruminococcaceae_V9D2013_group* on d 28, genus *unclassified_f_Ruminococcaceae*, *Anaerotruncus*, *Caproiciproducens*, and *Subdoligranulum* on d 42 was observed in WXY group. *Lachnospiraceae* has been demonstrated to produce butyrate; in addition, genus *norank_f_Lachnospiraceae, Shuttleworthia*, and *Ruminococcus_torques_group* have been observed to be related to better performance (Torok et al., 2011; Lee et al., 2017; McCafferty et al., 2019a; Li et al., 2020). Genera (*Hydrogenoanaerobacterium* and *Ruminococcaceae_V9D2013_group*) from the phylum Firmicutes are also suggested to effectively produce butyrate (Fang et al., 2020; Ilaria et al., 2020; Sun et al., 2020). Genus *unclassified_f_Ruminococcaceae*, *Anaerotruncus*, *Caproiciproducens*, and *Subdoligranulum* (family *Ruminococcaceae*) are also known as short-chain fatty acid producers. Supplemental xylanase could modulate GIT microbiota by increasing XOS concentrations in the cecum, and XOS has been reported to increase the abundance of butyrate-producing bacteria (De Maesschalck et al., 2015; McCafferty et al., 2019a). Moreover, the present result showed that xylanase supplementation to wheat-based diets increased the abundance of *Bifidobacterium* (phylum Actinobacteria) on d 14, and the abundance of *Lactobacillus* on d 42. XOS has been demonstrated to result in better performance of broilers by the stimulation of *Bifidobacterium* and *Lactobacillus* (Oyeagu et al., 2019; González-Ortiz et al., 2020). Although some species of Actinobacteria can lead to diseases, some strains like *Streptomyces* spp. exhibited antibacterial activities (Jing et al., 2018). *Bifidobacterium* as lactate-producing bacteria benefits to the health of GIT (Jing et al., 2018; González-Ortiz et al., 2020). Members of *Lactobacillus* could stimulate butyrate-producing bacteria and inhibit the colonization of pathogenic bacteria (Munyaka et al., 2016; Proszkowiec-Weglarz et al., 2020). When comparing cecal microbiota of broilers fed corn-based diets, compared with CCON, higher abundance of order *Clostridiales* on d 28, genus *Anaerofustis*, *Lachnospiraceae_UCG_010*, and *norank_f__Ruminococcaceae* on d 42 was observed in broilers from CXY group. Clostridium-related bacteria within GIT have been demonstrated to be able to produce short-chain fatty acids and act as a barrier against invasion by other potentially pathogenic microbiota (Munyaka et al., 2016). The increase of these butyrate-producing bacteria might also be related to increased XOS concentrations in the cecum.

Furthermore, compared with CCON group, lower abundance of family *Christensenellaceae*, *Desulfovibrionaceae*, and genus *Faecalitalea*, *Escherichia_Shigella* on d 14, and higher abundance of family *Erysipelotrichaceae* on d 42 were observed in CXY group. Xylanase supplementation to wheat-based diets could also increase the abundance of genus *Gordonibacter* on d 14 and d 42. The family *Christensenellaceae* was negatively associated with overall GIT health (Richards et al., 2019). *Faecalitalea* which affiliates to *Erysipelotrichaceae* family was related to inflammatory disorders (Dai et al., 2020). In addition, the previous work has reported that the abundance of *Desulfovibrio (*family *Desulfovibrionaceae)* was increased with ulcerative colitis, indicating that *Desulfovibrio* was enriched in an inflammatory environment (Yan et al., 2019). *Enterobacteriaceae* family includes potential pathogens, such as *Escherichia_Shigella*, the abundances of *Escherichia_Shigella* were found to be negatively correlated with growth performance in broilers (Díaz Carrasco et al., 2018). Moreover, *Erysipelotrichaceae* showed a positive correlation with growth performance (Stanley et al., 2016). *Gordonibacter* was associated with a decrease risk or severity of different diseases, such as cancer and inflammatory bowel disease (Pérez-Burillo et al., 2018). Overall, the results indicated that xylanase supplementation could stimulate the colonization of beneficial bacteria or inhibit the colonization of harmful bacteria in broilers receiving wheat- or corn-based diets.

Additionally, we compared the cecal microbiota of broilers supplemented corn- or wheat-based diets. The abundance of *Lactobacillus* and *Bifidobacterium* increased in the cecal microbiota of broilers receiving wheat-based diets, and the increase may be associated with high contents of AX in wheat. The cecal fermentative ability of broilers depends on type and content of substrates flowing into cecum. Members of *Lactobacillus* contained a range of genes coding ford AX-degrading enzymes; therefore, they can be beneficial to the degradation of XOS and AX (Zhang et al., 2018b). It also has been reported that AX and XOS selectively increased the abundance of *Bifidobacterium* spp., a so-called bifidogenic effect exerted by these prebiotics. Also, *Bifidobacterium* spp. have been reported to contain large number of genes coding for AX-degrading enzymes (Rivière et al., 2014). Overall, AX and XOS are preferred substrates of *Lactobacillus* and *Bifidobacterium*, and the growth of *Lactobacillus* and *Bifidobacterium* was stimulated by wheat relative to corn.

In corn-based diets fed broilers, the abundance of butyrate-producing bacterium increased, such as *Lachnospiraceae_UCG_010*, *Shuttleworthia*, *Anaerofilum*, *Ruminococcaceae_UCG_008*, *Ruminococcaceae_UCG_005*, *Ruminococcus_2*, *norank_f__Ruminococcaceae*, *Oscillibacter*, and *Ruminiclostridium_9* comparing to the broilers fed wheat-based diets. This difference may be attributed to the resistant starch content in corn and wheat. Corn contains the content of resistant starch 1.9-fold higher than that in wheat. Resistant starch is fermented by microbiota in large intestine and relates to increased abundance of butyrate-producing bacteria (Bednar et al., 2001; Yang et al., 2017b). Moreover, resistant starch-rich diet was associated with increased circulating propionate concentration in mice (Bai et al., 2021). In the current study, higher abundance of propionic acid-producing bacteria *Propionibacteriaceae* was likely to relate to the higher content of resistant starch in corn.

In wheat-based diets fed broilers, the abundance of Proteobacteria was higher than those fed corn-based diets, which may relate to proteinaceous substrates since wheat-based diets contain about 1% more protein than corn-based diets in this study. Proteobacteria are mainly responsible for utilization of amino acids and possess extensive gene coverage of amino acid responses (Dai et al., 2015; Diether and Willing, 2019). Moreover, the reason that higher abundance of *Peptostreptococcaceae* in the broilers fed corn-based diets was likely due to the different amino acid compositions of corn protein from wheat protein. Zein as the major fraction of corn protein is rich in leucine (Cowieson, 2005). *Peptostreptococcus spp*. was associated with leucine metabolism in hindgut (Ma and Ma, 2018).

## Conclusion

Overall, AT-xynA supplementation to wheat- or corn-based diets modulated the relative abundance of specific bacteria without changing the overall microbial structure. We showed that bacterial community clustering was mainly due to cereal grain source rather than xylanase supplementation. The addition of xylanase to wheat-based diets increased the abundance of *Lactobacillus*, *Bifidobacterium*, and some butyrate-producing bacteria, the increase might be related to the release of XOS as a result of AX degradation in GIT. Moreover, the undigested polysaccharides flowing into the cecum resulted in higher abundance of (non-starch polysaccharides) NSP-degrading bacteria in broilers fed wheat-based diets without xylanase supplementation, such as *Ruminococcaceae* and Bacteroidetes. Xylanase supplementation to corn-based diets slightly affected the abundance of butyrate-producing bacteria and NSP-degrading bacterium, the difference might be related to AX concentration and composition in corn and wheat. However, xylanase still increased the abundance of beneficial bacteria and decreased the abundance of harmful bacteria in the cecum of broilers fed corn-based diets. Moreover, beneficial effects of xylanase on cecal microbiome improved growth performance.

## Data Availability Statement

The datasets presented in this study can be found in online repositories. The names of the repository/repositories and accession number(s) can be found at: https://www.ncbi.nlm.nih.gov/, PRJNA753623.

## Ethics Statement

The animal study was reviewed and approved by the Laboratory Animal Welfare and Animal Experimental Ethical Inspection Committee of China Agricultural University.

## Author Contributions

YC and SL supervised and provided continuous guidance for the experiments. YC, BD, and CW conceived and designed the experiments. JW, HC, CB, and YL performed the animal experiments. JW, ZS, and BD analyzed the data and wrote the manuscript. All authors have discussed the results and reviewed the manuscript.

## Funding

Our study was supported by the funds of National Natural Science Foundation of People’s Republic of China (no. 31760673) and the Project of introducing talents to Tibet (RXYJ-2019-06).

## Conflict of Interest

The authors declare that the research was conducted in the absence of any commercial or financial relationships that could be construed as a potential conflict of interest.

## Publisher’s Note

All claims expressed in this article are solely those of the authors and do not necessarily represent those of their affiliated organizations, or those of the publisher, the editors and the reviewers. Any product that may be evaluated in this article, or claim that may be made by its manufacturer, is not guaranteed or endorsed by the publisher.

## Footnotes

^1^ http://drive5.com/uparse/

## References

[ref1] Bai Y. Li Y. Marion T. Tong Y. Zaiss M. M. Tang Z.. (2021). Resistant starch intake alleviates collagen-induced arthritis in mice by modulating gut microbiota and promoting concomitant propionate production. J. Autoimmun. 116:102564. doi: 10.1016/j.jaut.2020.102564, PMID: 33203617

[ref2] Bautil A. Verspreet J. Buyse J. Goos P. Bedford M. R. Courtin C. M. (2019). Age-related arabinoxylan hydrolysis and fermentation in the gastrointestinal tract of broilers fed wheat-based diets. Poult. Sci. 98, 4606–4621. doi: 10.3382/ps/pez159, PMID: 30993340

[ref3] Bednar G. E. Patil A. R. Murray S. M. Grieshop C. M. Merchen N. R. Fahey G. C. Jr. (2001). Starch and fiber fractions in selected food and feed ingredients affect their small intestinal digestibility and fermentability and their large bowel fermentability in vitro in a canine model. J. Nutr. 131, 276–286. doi: 10.1093/jn/131.2.276, PMID: 11160546

[ref4] Biddle A. Stewart L. Blanchard J. Leschine S. (2013). Untangling the genetic basis of fibrolytic specialization by Lachnospiraceae and Ruminococcaceae in diverse gut communities. Diversity 5, 627–640. doi: 10.3390/d5030627

[ref5] Cao Y. H. Qiao J. Y. Li Y. H. Lu W. Q. (2007). De novo synthesis, constitutive expression of aspergillus sulphureus β-xylanase gene in Pichia pastoris and partial enzymic characterization. Appl. Microbiol. Biotechnol. 76, 579–585. doi: 10.1007/s00253-007-0978-9, PMID: 17646981

[ref6] Chakraborti C. K. (2015). New-found link between microbiota and obesity. World J. Gastrointest. Pathophysiol. 6:110. doi: 10.4291/wjgp.v6.i4.110, PMID: 26600968 PMC4644874

[ref7] Choct M. Hughes R. J. Bedford M. R. (1999). Effects of a xylanase on individual bird variation, starch digestion throughout the intestine, and ileal and caecal volatile fatty acid production in chickens fed wheat. Brit. Poult. Sci. 40, 419–422. doi: 10.1080/00071669987548 10475642

[ref8] Cowieson A. J. (2005). Factors that affect the nutritional value of maize for broilers. Anim. Feed Sci. Technol. 119, 293–305. doi: 10.1016/j.anifeedsci.2004.12.017

[ref9] Cowieson A. J. (2010). Strategic selection of exogenous enzymes for corn/soy-based poultry diets. J. Poult. Sci. 47, 1–7. doi: 10.2141/jpsa.009045

[ref10] Dai D. Qiu K. Zhang H. Wu S. Han Y. Wu Y.. (2021). Organic acids as alternatives for antibiotic growth promoters alter the intestinal structure and microbiota and improve the growth performance in broilers. Front. Microbiol. 11:618144. doi: 10.3389/fmicb.2020.618144 33519778 PMC7840962

[ref11] Dai Z. Wu Z. Hang S. Zhu W. Wu G. (2015). Amino acid metabolism in intestinal bacteria and its potential implications for mammalian reproduction. Mol. Hum. Reprod. 21, 389–409. doi: 10.1093/molehr/gav003, PMID: 25609213

[ref12] Dai D. Wu S. Zhang H. Qi G. Wang J. (2020). Dynamic alterations in early intestinal development, microbiota and metabolome induced by in ovo feeding of L-arginine in a layer chick model. J. Anim. Sci. Biotechnol. 11:19. doi: 10.1186/s40104-020-0427-5 32175081 PMC7063725

[ref13] De Maesschalck C. Eeckhaut V. Maertens L. De Lange L. Marchal L. Nezer C.. (2015). Effects of xylo-oligosaccharides on broiler chicken performance and microbiota. Appl. Environ. Microb. 81, 5880–5888. doi: 10.1128/AEM.01616-15, PMID: 26092452 PMC4551243

[ref14] Dhillon J. Li Z. Ortiz R. M. (2019). Almond snacking for 8 wk increases alpha-diversity of the gastrointestinal microbiome and decreases Bacteroides fragilis abundance compared with an isocaloric snack in college freshmen. Curr. Dev. Nutr. 3:z79. doi: 10.1093/cdn/nzz079 PMC6736066 31528836

[ref15] Díaz Carrasco J. M. Redondo E. A. Pin Viso N. D. Redondo L. M. Farber M. D. Fernández Miyakawa M. E. (2018). Tannins and bacitracin differentially modulate gut microbiota of broiler chickens. Biomed. Res. Int. 2018, 1–11. doi: 10.1155/2018/1879168, PMID: 29682522 PMC5841071

[ref16] Diether N. E. Willing B. P. (2019). Microbial fermentation of dietary protein: an important factor in diet-microbe-host interaction. Microorganisms. 7:19. doi: 10.3390/microorganisms7010019, PMID: 30642098 PMC6352118

[ref17] Fang S. Chen X. Pan J. Chen Q. Zhou L. Wang C.. (2020). Dynamic distribution of gut microbiota in meat rabbits at different growth stages and relationship with average daily gain (ADG). BMC Microbiol. 20:116. doi: 10.1186/s12866-020-01797-5 32410629 PMC7227296

[ref18] González-Ortiz G. Olukosi O. A. Jurgens G. Apajalahti J. Bedford M. R. (2020). Short-chain fatty acids and ceca microbiota profiles in broilers and turkeys in response to diets supplemented with phytase at varying concentrations, with or without xylanase. Poult. Sci. 99, 2068–2077. doi: 10.1016/j.psj.2019.11.051, PMID: 32241492 PMC7587645

[ref19] Gonzalez-Ortiz G. Sola-Oriol D. Martinez-Mora M. Perez J. F. Bedford M. R. (2017). Response of broiler chickens fed wheat-based diets to xylanase supplementation. Poult. Sci. 96, 2776–2785. doi: 10.3382/ps/pex092, PMID: 28431146

[ref20] Ilaria B. Ilario F. Sihem D. Rocchina E. Francesco G. Laura G.. (2020). Black soldier fly and gut health in broiler chickens: insights into the relationship between cecal microbiota and intestinal mucin composition. J. Anim. Sci. Biotechnol. 11:11. doi: 10.1186/s40104-019-0413-y, PMID: 32025297 PMC6996183

[ref21] Jing Y. Li A. Liu Z. Yang P. Wei J. Chen X.. (2018). Absorption of Codonopsis pilosula saponins by coexisting polysaccharides alleviates gut microbial dysbiosis with dextran sulfate sodium-induced colitis in model mice. Biomed. Res. Int. 2018, 1–18. doi: 10.1155/2018/1781036, PMID: 30211217 PMC6120299

[ref22] Khadem A. Lourenco M. Delezie E. Maertens L. Goderis A. Mombaerts R.. (2016). Does release of encapsulated nutrients have an important role in the efficacy of xylanase in broilers? Poult. Sci. 95, 1066–1076. doi: 10.3382/ps/pew002, PMID: 26908893

[ref23] Kiarie E. Romero L. F. Ravindran V. (2014). Growth performance, nutrient utilization, and digesta characteristics in broiler chickens fed corn or wheat diets without or with supplemental xylanase. Poult. Sci. 93, 1186–1196. doi: 10.3382/ps.2013-03715, PMID: 24795311

[ref24] Lee K. Kil D. Y. Sul W. J. (2017). Cecal microbiome divergence of broiler chickens by sex and body weight. J. Microbiol. 55, 939–945. doi: 10.1007/s12275-017-7202-0, PMID: 29214491

[ref25] Li H. Zhang H. Zhao F. Wang S. Wang Z. Wei Z. (2020). Modulation of gut microbiota, short-chain fatty acid production, and inflammatory cytokine expression in the cecum of porcine Deltacoronavirus-infected chicks. Front. Microbiol. 11:897. doi: 10.3389/fmicb.2020.00897 32582042 PMC7287039

[ref26] Liu J. Stewart S. N. Robinson K. Yang Q. Lyu W. Whitmore M. A.. (2021). Linkage between the intestinal microbiota and residual feed intake in broiler chickens. J. Anim. Sci. Biotechnol. 12:22. doi: 10.1186/s40104-020-00542-2 33573700 PMC7879522

[ref27] Liu W. C. Kim I. H. (2017). Effects of dietary xylanase supplementation on performance and functional digestive parameters in broilers fed wheat-based diets. Poult. Sci. 96, 566–573. doi: 10.3382/ps/pew258, PMID: 27566730

[ref28] Ma N. Ma X. (2018). Dietary amino acids and the gut-microbiome-immune axis: physiological metabolism and therapeutic prospects. Compr. Rev. Food Sci. Food Saf. 18, 221–242. doi: 10.1111/1541-4337.12401 33337014

[ref29] Ma Z. Y. Zhang X. M. Wang M. Wang R. Jiang Z. Y. Tan Z. L.. (2019). Molecular hydrogen produced by elemental magnesium inhibits rumen fermentation and enhances methanogenesis in dairy cows. J. Dairy Sci. 102, 5566–5576. doi: 10.3168/jds.2018-15647, PMID: 30981486

[ref30] Masey-O'Neill H. V. Singh M. Cowieson A. J. (2014). Effects of exogenous xylanase on performance, nutrient digestibility, volatile fatty acid production and digestive tract thermal profiles of broilers fed on wheat- or maize-based diet. Brit. Poult. Sci. 55, 351–359. doi: 10.1080/00071668.2014.898836 24579789

[ref31] McCafferty K. W. Bedford M. R. Kerr B. J. Dozier W. A. (2019a). Effects of age and supplemental xylanase in corn- and wheat-based diets on cecal volatile fatty acid concentrations of broilers. Poult. Sci. 98, 4787–4800. doi: 10.3382/ps/pez194 31065717

[ref32] McCafferty K. W. Bedford M. R. Kerr B. J. Dozier W. A. (2019b). Effects of cereal grain source and supplemental xylanase concentrations on broiler growth performance and cecal volatile fatty acid concentrations from 1 to 40 d of age. Poult. Sci. 98, 2866–2879. doi: 10.3382/ps/pez032 30805626

[ref33] Munyaka P. M. Nandha N. K. Kiarie E. Nyachoti C. M. Khafipour E. (2016). Impact of combined beta-glucanase and xylanase enzymes on growth performance, nutrients utilization and gut microbiota in broiler chickens fed corn or wheat-based diets. Poult. Sci. 95, 528–540. doi: 10.3382/ps/pev333, PMID: 26574039

[ref34] National Research Council (1994). Nutrient Requirements of Poultry. 9th *Edn.* Washington, DC: The National Academies Press.

[ref35] Ocejo M. Oportp B. Hurtado A. (2019). 16S rRNA amplicon sequencing characterization of caecal microbiome composition of broilers and free-range slow-growing chickens throughout their productive lifespan. Sci. Rep. 9:2506. doi: 10.1038/s41598-019-39323-x 30792439 PMC6385345

[ref36] Onrust L. Ducatelle R. Van Driessche K. De Maesschalck C. Vermeulen K. Haesebrouck F.. (2015). Steering endogenous butyrate production in the intestinal tract of broilers as a tool to improve gut health. Front. Vet. Sci. 2:75. doi: 10.3389/fvets.2015.00075, PMID: 26734618 PMC4682374

[ref37] Oyeagu C. E. Mlambo V. Muchenje V. Marume U. (2019). Effect of dietary supplementation of aspergillus xylanase on broiler chickens performance. Iranian J. Appl. Anim. Sci. 9, 693–708.

[ref38] Pérez-Burillo S. Pastoriza S. Jiménez-Hernández N. Auria D. G. Francino M. P. Rufián-Henares J. A. (2018). Effect of food thermal processing on the composition of the gut microbiota. J. Agr. Food Chem. 66, 11500–11509. doi: 10.1021/acs.jafc.8b04077 30346155

[ref39] Petra V. Karel S. Maja L. Marcela F. Lenka G. Darina C.. (2014). Succession and replacement of bacterial populations in the caecum of egg laying hens over their whole life. PLoS One 9:e115142. doi: 10.1371/journal.pone.0115142, PMID: 25501990 PMC4264878

[ref40] Petry A. L. Patience J. F. Nichole F. H. Koester L. R. Bedford M. R. Schmitz-Esser S. (2021). Xylanase supplementation modulates the microbiota of the large intestine of pigs fed corn-based fiber by means of a stimbiotic mechanism of action. Front. Microbiol. 12:619970. doi: 10.3389/fmicb.2021.619970 33841350 PMC8024495

[ref41] Polansky O. Sekelova Z. Faldynova M. Sebkova A. Sisak F. Rychlik I. (2016). Important metabolic pathways and biological processes expressed by chicken cecal microbiota. Appl. Environ. Microb. 82, 1569–1576. doi: 10.1128/AEM.03473-15 PMC4771310 26712550

[ref42] Proszkowiec-Weglarz M. Miska K. B. Schreier L. L. Grim C. J. Jarvis K. G. Shao J.. (2020). Effect of butyric acid glycerol esters on ileal and cecal mucosal and luminal microbiota in chickens challenged with Eimeria maxima. Poult. Sci. 99, 5143–5148. doi: 10.1016/j.psj.2020.06.022, PMID: 32988553 PMC7598111

[ref43] Richards P. Fothergill J. Bernardeau M. Wigley P. (2019). Development of the caecal microbiota in three broiler breeds. Front. Vet. Sci. 6:201. doi: 10.3389/fvets.2019.00201 31294039 PMC6603203

[ref44] Ríos-Covián D. Ruas-Madiedo P. Margolles A. Gueimonde M. de Los Reyes-Gavilán C. G. Salazar N. (2016). Intestinal short chain fatty acids and their link with diet and human health. Front. Microbiol. 7:185. doi: 10.3389/fmicb.2016.00185, PMID: 26925050 PMC4756104

[ref45] Rivière A. Moens F. Selak M. Maes D. Weckx S. De Vuyst L. (2014). The ability of Bifidobacteria to degrade arabinoxylan oligosaccharide constituents and derived oligosaccharides is strain dependent. Appl. Environ. Microb. 80, 204–217. doi: 10.1128/AEM.02853-13, PMID: 24141124 PMC3911024

[ref46] Shang Y. Kumar S. Oakley B. Kim W. K. (2018). Chicken gut microbiota: importance and detection technology. Front. Vet. Sci. 5:254. doi: 10.3389/fvets.2018.00254 30406117 PMC6206279

[ref47] Stanley D. Hughes R. J. Geier M. S. Moore R. J. (2016). Bacteria within the gastrointestinal tract microbiota correlated with improved growth and feed conversion: challenges presented for the identification of performance enhancing probiotic bacteria. Front. Microbiol. 7:187. doi: 10.3389/fmicb.2016.00187, PMID: 26925052 PMC4760072

[ref48] Sun X. Shen J. Liu C. Li S. Peng Y. Chen C.. (2020). L-arginine and N-carbamoylglutamic acid supplementation enhance young rabbit growth and immunity by regulating intestinal microbial community. Asian Austral. J. Anim. 33, 166–176. doi: 10.5713/ajas.18.0984 PMC6946986 31208171

[ref49] Torok V. A. Hughes R. J. Mikkelsen L. L. Perez-Maldonado R. Balding K. MacAlpine R.. (2011). Identification and characterization of potential performance-related gut microbiotas in broiler chickens across various feeding trials. Appl. Environ. Microb. 77, 5868–5878. doi: 10.1128/AEM.00165-11, PMID: 21742925 PMC3165380

[ref50] Wang J. Liu Y. Yang Y. Bao C. Cao Y. (2020). High-level expression of an acidic thermostable xylanase in Pichia pastoris and its application in weaned piglets. J. Anim. Sci. 98:skz364. doi: 10.1093/jas/skz364 31778535 PMC6986428

[ref51] Wang J. Liu S. J. Ma J. Y. Piao X. S. (2021). Changes in growth performance and Ileal microbiota composition by Xylanase supplementation in broilers fed wheat-based diets. Front. Microbiol. 12:706396. doi: 10.3389/fmicb.2021.706396 34335542 PMC8319766

[ref52] Yadav S. Jha R. (2019). Strategies to modulate the intestinal microbiota and their effects on nutrient utilization, performance, and health of poultry. J. Anim. Sci Biotechnol. 10:2. doi: 10.1186/s40104-018-0310-9 30651986 PMC6332572

[ref53] Yan J. Zhou B. Xi Y. Huan H. Li M. Yu J.. (2019). Fermented feed regulates growth performance and the cecal microbiota community in geese. Poult. Sci. 98, 4673–4684. doi: 10.3382/ps/pez169, PMID: 30993344

[ref54] Yang J. Martinez I. Walter J. Keshavarzian A. Rose D. J. (2013). In vitro characterization of the impact of selected dietary fibers on fecal microbiota composition and short chain fatty acid production. Anaerobe 23, 74–81. doi: 10.1016/j.anaerobe.2013.06.012, PMID: 23831725

[ref55] Yang W. Yang Y. Zhang L. Xu H. Guo X. Yang X.. (2017a). Improved thermostability of an acidic xylanase from aspergillus sulphureus by combined disulphide bridge introduction and proline residue substitution. Sci. Rep. 7:1587. doi: 10.1038/s41598-017-01758-5 28484256 PMC5431495

[ref56] Yang X. Darko K. O. Huang Y. He C. Yang H. He S.. (2017b). Resistant starch regulates gut microbiota: structure, biochemistry and cell signalling. Cell. Physiol. Biochem. 42, 306–318. doi: 10.1159/000477386 28535508

[ref57] Zhang Z. Tun H. M. Li R. Gonzalez B. J. M. Keenes H. C. Nyachoti C. M.. (2018a). Impact of xylanases on gut microbiota of growing pigs fed corn- or wheat-based diets. Anim. Nutr. 4, 339–350. doi: 10.1016/j.aninu.2018.06.007 30564753 PMC6284322

[ref58] Zhang Y. J. Liu Q. Zhang W. M. Zhang Z. J. Wang W. L. Zhuang S. (2018b). Gastrointestinal microbial diversity and short-chain fatty acid production in pigs fed different fibrous diets with or without cell wall-degrading enzyme supplementation. Livest. Sci. 207, 105–116. doi: 10.1016/j.livsci.2017.11.017

